# Mediating roles of perceived social support and hopelessness in the relationship between negative life events and self-identity acquisition among Chinese college students with left-behind and/or migrant experiences

**DOI:** 10.3389/fpsyg.2025.1530107

**Published:** 2025-03-07

**Authors:** Lijuan Liu, Jingbo Li, Yanlan Li

**Affiliations:** ^1^School of Literature and Journalism, Yichun University, Yichun, China; ^2^School of Physical Science and Technology, Yichun University, Yichun, China; ^3^School of Normal Education, Yichun University, Yichun, China

**Keywords:** left-behind experiences, left-behind-migrant experiences, self-identity acquisition, negative life events, perceived social support, hopelessness, college students

## Abstract

**Introduction:**

Although research examining the status of self-identity of college students has been conducted, few studies have explored the intrinsic mechanisms underlying the formation of self-identity acquisition (SIA) among college students with experiences of being left behind and/or migrant. The acquisition of self-identity is a crucial task during the college years, becoming even more significant in the aftermath of stressful life events.

**Methods:**

This study employed Self-identity Acquisition (SIA), College Students Self-Rating Life Events Checklist, Perceived Social Support Scale (PSSS), Hopelessness scale to explore the impact of negative life events on the acquisition of self-identity among college students with left-behind and/or migrant experiences. A total of 1,240 college students were surveyed, comprising 180 with both left behind and migrant experiences (Group 1), 556 with only left-behind experiences (Group 2), 117 with migrant experiences (Group 3), and 387 with no left-behind or migrant experiences (Group 4).

**Results:**

Students in Group 1 exhibited the lowest levels of SIA, followed by those in Group 2, while participants in Group 4 demonstrated superior outcomes. The results of the correlation analysis showed that four variables were significantly correlated exclusively within the two groups of students experiencing left behind (Group 1 and Group 2), meeting the conditions for conducting a mediation test. In Group 2, perceived social support (PSSS) and hopelessness played separate mediating roles between negative life events and SIA. However, for individuals who experienced both left behind and migrant situations, there was no significant mediating effect of PSSS between negative life events and SIA; instead, hopelessness served as a stronger mediator compared to its role within the left-behind group. The two groups characterized by left-behind experiences, PSSS and hopelessness play chain mediating roles.

**Discussion:**

These findings suggest that such dual experiences may lead to an increased perception of available support while simultaneously fostering despair that hinders the development toward acquiring a solid sense of identity. When individuals with left-behind experiences encounter feelings associated with despair following adverse life events, they tend to diminish their utilization of available supportive resources, which is not conducive to making positive and firm self-identity exploration and commitment.

## 1 Introduction

### 1.1 Left-behind or/and migration

With the rapid advancement of industrialization and urbanization in China, the phenomenon of rural-to-urban internal migration has become increasingly prevalent, giving rise to a substantial floating population. Data from the Seventh National Population Census in 2021 indicate that China’s floating population has surged to 376 million (National Bureau of Statistics, 2021). Owing to China’s household registration system and the economic status of migrants themselves, children of migrants can generally be classified into two major categories: left-behind children and migrant children (National Women's Federation Research Group, 2013). Left-behind children are those who have been separated from both parents or one parent for over 6 months before turning 16 and are entrusted to the care of grandparents or other guardians due to their parents’ absence for work or business purposes in another location; Migrant children refer to individuals who departed from their registered residence before turning 16 and relocated with both parents or one parent for more than 6 months (National Women's Federation Research Group, 2013). According to statistical data from the “China Rural Education Development Report 2020–2022″, as of late 2021, there are approximately 11.992 million left-behind children in rural China and about 13.724 million migrant children, totaling around 25.716 million (People’s Daily, 2022). Compared with previous years, numbers for both groups have decreased; however, they remain significant and noteworthy (National Women's Federation Research Group, 2013; Ministry of Education, 2018).

For left-behind children, while they may benefit from the improved economic investment made by their migrant parents in aspects such as materials, education, and healthcare, fewer have achieved a stable sense of self-identity. The majority find themselves experiencing delayed or diffused self-identities (Chen, 2023). Migrant children generally possess a relatively intact family structure and do not have psychological issues stemming from parent–child separation. Furthermore, they have the opportunity to broaden their horizons, make new friends, and access local social resources in urban areas. However, they may encounter social exclusion and discrimination due to structural barriers associated with the absence of local household registration, which can result in an uncertain or chaotic state of self-identity (Liu and Jin, 2018). It is important to note that another group of children exists who have experienced both being left behind and migrating. Previous research has shown that these children perceive significant differences between themselves and their urban counterparts; they often exhibit an emotional inclination toward their rural identity while frequently finding themselves in a state of contradiction and confusion regarding their identity attribution (Deng and Chen, 2017).

Previous research has indicated that the influence of childhood experiences on individuals persists into adulthood (Liu, 2021; Fan et al., 2009). Some studies discovered the adverse effects of being left behind during childhood can continue to impact an individual’s self-identity and cognitive evaluation well into adulthood (Han, 2018; Xie, 2017; Liu, 2021). Conversely, other researchers (Wang, 2015; Xie and Zhang, 2011) argued that most individuals who experienced being left behind can choose activities they desire based on their own will and engage in them with a positive attitude. During the university stage, they may develop proactive exploration and selection skills. As such, it is suggested that the “left-behind experience” can serve as a positive influencing factor for personal development. The migrant experience during childhood also affects future development. Thomas (2016) presented that the development of the identity of migrant individuals may become potentially unstable due to changes in time and region (Schubach et al., 2016). However, other studies have found that mobility might serve as a protective factor (Zhang et al., 2015). There appeared to be no statistically significant difference in its impact on an individual’s self-evaluation and social adaptation in adulthood when compared to those who did not experience moving or being left behind (Xie, 2017; Deng and Chen, 2017). At the same time, some literature indicated that having both left-behind and migrant experiences can have a more detrimental impact on an individual’s development. Upon entering college, they often exhibit poorer performance regarding core self-evaluation, mental health, etc. (Xie, 2017), making them prone to issues related to self-identity such as “who I am” and “what kind of person I can become.” To sum up, it remains uncertain how early left-behind or migrant experiences influence self-identity development among college students. Therefore, this study aims to further explore the state of self-identity among those who were left behind and/or mobility during childhood as they grow up, to comprehend the long-term effects of their childhood experiences of being left behind and/or migrating.

A considerable number of prior studies have concentrated on the comparisons between college students who were once left behind in rural areas and their peers, investigating the identity status of these individuals. Empirical research conducted by Luo et al. (2021) revealed that the physiological self, psychological self, family self, self-concept, self-satisfaction, and self-identity among college students who experienced being left behind were lower than those of their counterparts without such experiences (Chen and Zhang, 2017); but, they demonstrated higher levels of role slack and role conflict (Liu, 2018). Nevertheless, there have been scarce studies investigating the similarities and differences in identity between college students with migratory experiences and their peers. A few studies have discovered that college students with migratory backgrounds exhibited more positive core self-evaluations and employed more effective coping strategies compared to those who were previously left behind; however, no significant differences were observed when compared to ordinary college students (Zhang et al., 2015; Xie, 2017). Nonetheless, contrastive studies specifically addressing self-identity remain lacking. Furthermore, research on college students who have had both migratory and left-behind experiences is even more limited. One article conducted by Xie (2017) investigated this group and indicated that their core self-evaluation and mental health conditions were notably poorer compared to three other groups. However, this research failed to discuss the self-identity of this group.

Given the unique context of China’s social development, it is likely that issues related to rural left-behind children and migrant youth will persist for an extended period. This raises critical questions about whether to stay in one place, migrate, or combine both, yields better developmental outcomes for children. This paper classifies college students into four distinct groups based on their past experiences of being left behind or migrating and conducts comparative analyses among these groups. It is hoped that the results of our research will provide valuable insights for parental decision-making and inform the development of educational policies by government authorities.

### 1.2 Self-identity

Identity is an intrinsic and self-constructed framework. The establishment and maintenance of self-identity commitments represent a continuous effort in which individuals consistently form, evaluate, and adjust their existing commitments (Hasanshahi et al., 2018). According to the two primary process variables in Erikson’s theory of identity—degrees of exploration and commitment, Marcia (1980b) divided self-identity into four distinct states—acquisition, diffusion, foreclosure, and moratorium. Among these, the achievement of self-identity signifies that an individual has a consecutive and coherent subjective awareness and experience regarding their abilities, beliefs, and values. That state forms a commitment to future endeavors based on active exploration of the questions “who I am” and “what my path is for future development” (Erikson, 1968; Marcia, 1966). In short, drawing from Marcia (1980), we argue that individuals who achieve identity have engaged in exploration, carefully considered various options, and made strong commitments to specific goals, beliefs, and values.

According to developmental contextualism theory (Zhang and Chen, 2009), the development of self-identity is achieved through ongoing interactions between the individual and the surrounding context. Among college students, a sense of self-identity emerges across multiple life domains, with educational and interpersonal relationships being two fundamental and highly prominent domains (Schwartz et al., 2011). The domain of educational identity encompasses commitment to learning and related academic activities. Within academic settings, many young individuals demonstrate learning motivation in various subjects and make significant decisions regarding their career orientations. In terms of interpersonal identity, Grotevant et al. (1982)asserted that support from peers and parents provides a context for young people to explore and develop their self-awareness, thereby facilitating the formation of self-identity while offering a background for commitments to parents or friends.

Research conducted by Meeus et al. (2010) discovered that during adolescence, individuals’ exploration behaviors tend to decrease as commitments increase; furthermore, self-identification exhibits a high degree of stability over time. However, Meilman (1979) found that individuals typically struggle to establish a consistent sense of identity before the age of 18. It is often not until the university stage that many individuals transition from states of diffusion and foreclosure to moratorium status, ultimately achieving a solid sense of identity (Kroger, 2007). Cramer (1998) noted that during late adolescence, specifically throughout the college years, more than half of individuals persist in states characterized by low exploration despite transitions toward higher exploration being possible. Additionally, Goossens et al. revealed that 50–73% of college students were in exploration processes while only 10–30% had made firm commitments (Goossens, 2001; Wang et al., 2007; Zhou et al., 2024). Overall, a relatively small number of college students have actively engaged in exploration and made definitive commitments. In other words, the proportion of individuals who successfully attain a sense of self-identity is relatively low.

During their college years, students are liberated from parental strictures and the constraints imposed by exam-oriented education. They gain autonomy over their lives and studies, allowing them to explore various options, reposition themselves, and contemplate issues related to self-identity such as “who I am” and “how I became this person.” A well-developed sense of self-identity enables individuals to gain a clearer awareness of their own strengths and weaknesses (Marcia, 1980b). By cultivating a robust commitment to their identity, young people can acquire a sense of continuity and meaning in life (Erikson, 1968). Failure to achieve satisfactory development in self-identity can lead to challenges including unclear cognition of both present and future selves, low self-esteem (Chen, 2023), self-doubt, and identity crisis (Han, 2018; Wang, 2019). Moreover, individuals may be more susceptible to exhibiting negative emotional issues such as anxiety and depression (Kroger, 2007), along with potential anti-social behaviors (Erikson, 1968), suicidal ideation, and suicidal behaviors (Ying, 2013). Therefore, engaging in self-exploration while establishing a coherent sense of self-identity is regarded as a significant task for adolescents and young adults.

These prior studies on human-centered identity development have emphasized individual differences within their process (Meeus et al., 2010b). This underscores the necessity to investigate predictors influencing self-identity development among individuals. Specific life events may trigger distinct states concerning one’s identity; such changes often become more prominent following stressful life events (Anthis, 2002). Therefore, it is crucial to examine how these events impact college students’ acquisition of self-identity.

### 1.3 Self-identity and negative life events

The college stage represents the latter phase in adolescent development—a transitional period before stepping into society. During this time, they need to navigate various changes, including the loss of relatives or friends, academic achievements, or employment pressures. Generally speaking, the primary sources of stress for college students stem from interpersonal relationships and academic demands (Liu and Li, 2004). Nearly 60% of European adolescents reported having lost a friend or family member within the past year (Madge et al., 2011). Among normal university students, 79.41% indicated that they had experienced disagreements with their classmates or friends, 85.78% reported feeling overwhelmed by their study load, and 60.29% expressed that they were under significant academic pressure (Liu and Li, 2004).

The experience of stressful events is recognized as a significant factor in highlighting the importance of identity. Negative stressful events have been shown to significantly hinder the acquisition of self-identity; specifically, the higher the level of negative stressful events, the lower the status of self-identity acquisition (Yang, 2017). Furthermore, these adverse events even change levels of exploration and commitment, resulting in diffusion or delays of self-identity. Stressful life events act as external factors that may disrupt an individual’s internal psychological resources, resulting in a loss of self-identity and continuity (Erikson, 1968). This discontinuity, in turn, may force individuals to reassess their current life situation by integrating their past experiences into their sense of self (Anthis, 2002), engaging in behaviors that are detrimental to the construction and maintenance of identity commitment (Grotevant, 1987). Indeed, stressful events can cause individuals to face challenges related to psychological difficulties, and pre-existing psychological difficulties may amplify the impacts of such stressors (Sutherland and Bryant, 2005). Research indicated that these stressful events serve as pivotal turning points that can impact an adolescent’s capacity to establish a coherent sense of self at a psychological level (Ionio et al., 2013). And it is a crucial point in forming a cohesive self (Habermas and Bluck, 2000) and a clear identity (Ogle et al., 2013).

Being exposed to stressful life events can lead to a weakening of commitment. A study conducted by Kroger and Green (1996) involving adults revealed that individuals who experienced the loss of a family member or friend demonstrated lower levels of exploration, often either adhering to their current commitments or reverting to diffusion. Longitudinal survey data indicated that adolescents encountering more negative life events exhibited weaker career commitment 3 years later (van Doeselaar et al., 2018). Updegraff and Taylor (2000) further illustrated that interpersonal commitment may diminish following stressful life events. In summary, based on life events and identity theory, adolescents may exhibit weaker commitments after experiencing negative life events.

However, van Doeselaar et al. (2018) conducted two longitudinal studies involving Dutch adolescents and young adults, spanning 8 and 6 years, respectively. Their findings revealed that individuals’ interpersonal commitment remained unaffected after experiencing negative life events. Additionally, in a five-year investigation, Schubach et al. (2016) discovered that adolescents were actively engaged in the process of self-identity construction. These adolescents adjusted their self-perceptions regardless of whether they encountered stressful life events; consequently, such experiences had no significant impact on the development of their self-identity (De Moor et al., 2019).

To sum up, it is found that the influence of stressful life events on the development of self-identity remains uncertain. Therefore, this study attempts to further explore the relationship between stressful life events and the acquisition of self-identity among college students. We hope to provide a reference for the formation of self-identity for college students who have experienced left-behind and/or transient circumstances during their early years. Thus, we propose hypothesis 1 (H1): negative life events negatively predict self-identity acquisition.

### 1.4 The mediating role of perceived social support

Previous studies have identified both inter-individual social relationships and intra-individual factors that influence adolescents’ adaptation to the effects of stressful life events (De Moor et al., 2019; Yang et al., 2010). The characteristics of social relationships, including support from family and friends, can mitigate the impact of stressors on mental health (Yang et al., 2010), provide an environment for adolescents to explore and develop self-awareness, and facilitate the formation of self-identity (Becht et al., 2017). In accordance with the social support mitigation model (Huber et al., 2021), such support plays a buffering role against the detrimental effects of negative life events. It can enhance the individual’s resilience and adaptability while promoting levels of exploration and commitment. Research on threatening or hostile traumatic family experiences has shown that these incidents can affect an individual’s state of self-identity through mediation by social support (Yuan, 2021).

During the university stage, individuals face challenges in developing a coherent sense of self and establishing their positions within social groups. Stressful events encountered in academic and daily life can exacerbate negative emotions, which may manifest as adverse evaluations of others and the surrounding environment, thereby reducing an individual’s perception of available support (Zhou et al., 2022). At this time, emotional and social support from family and friends becomes essential. Family members and friends are vital sources of companionship and support during adolescence (Scholte et al., 2001). They can buffer the adverse effects associated with negative life events, thereby helping adolescents develop a coherent sense of self (Becht et al., 2017; Ji et al., 2023). A survey conducted by Wang (2007) involving 1,131 college students indicated that high levels of trust and communication, along with low alienation in the parent–child relationship, constitute an optimal relationship environment for the development of adolescents. This environment contributes to the formation and evolution of self-identity among college students. In comparison to other states related to ego identity development, adolescents in the achieved state exhibited the highest level of parental attachment (Benson et al., 1992). Similarly, Meeus et al. (2002a) discovered that trust in peer attachments was also beneficial for college students’ commitment to their identities; effective communication and support from peers predict higher levels of identity exploration.

Communication and trust between parents and children or among peers are subjective feelings and support for individuals. The emotional support experienced subjectively can significantly predict the development of self-identity (Li et al., 2011). The stronger the perception of support from parents and friends among college students, the more positively they engage in self-involvement (Yan, 2013). Therefore, this study proposes hypothesis 2 (H2): perceptive social support plays a mediating role between negative life events and self-identity acquisition among college students.

### 1.5 The mediating role of hopelessness

Hopelessness is characterized as a negative self-perceptual schema and an adverse expectation for the future (Yang, 2013). Beck emphasized that despair may arise from the activation of an individual’s specific self-perception system and cognitive schema. When triggered by negative life events, individuals often modify their expectations regarding themselves and their futures, interpret their experiences in a pessimistic light, and become engulfed in feelings of pessimism and hopelessness. An empirical study (Yang, 2013) revealed that 73.5% of college students had different degrees of despair, with 22% exhibiting moderate or higher levels of this condition. Those who reported a high sense of despair have typically encountered more adverse life events and endured greater stress. Negative life events directly influence the formation of despair among college students (Kim et al., 2016). When individuals attribute these unfavorable occurrences to internal and stable causes, they are prone to engage in self-blame, lose confidence in their future, and allow the detrimental effects of such events to permeate all aspects of their lives. This tendency ultimately generates a profound sense of despair (Yang, 2013).

Anxiety, disappointment, and despair are prevalent issues that college students face in the process of establishing their self-identity. These emotions can significantly influence the construction of self-identity and ongoing self-identification (Zhang, 2017; Sokol and Serper, 2017). When individuals adopt a desperate cognitive style and perceive their future as hopeless, their patterns of thinking and solving problems tend to become increasingly negative, and their goal orientation will weaken (Sokol and Serper, 2017; Guo and Wu, 2011). Consequently, they may consider that their present selves to their future selves are deteriorating, resulting in a more pessimistic outlook on their future selves (Sokol and Serper, 2017).

Negative life events, if not adequately addressed, can lead individuals to be mired in negative emotions for a prolonged period. This prolonged emotional state leaves little room for the exploration and development of self-identity. Individuals with high levels of negative emotions are more susceptible to undergoing stressful life events (Yang, 2013), which further exacerbates the crisis surrounding their self-identity (Zhang, 2017; Sokol and Serper, 2017). On the contrary, individuals with fewer academic challenges typically exhibit healthier coping strategies. They are less likely to remain trapped in negative emotional states for a long time (Li, 2018). As a result, they can be better equipped to confront the challenges associated with the process of self-identity development. So, we propose hypothesis 3 (H3): hopelessness mediates the relationship between negative life events and college students’ self-identity.

### 1.6 The chain-mediated role of perceived social support and hopelessness

Based on the above analysis, it is proposed that perceived social support and hopelessness may serve as mediators between negative life events and college students’ self-identity. The acquisition of self-identity is not an automatic process; rather, it requires effort during the process of becoming, necessitating both external support and internal psychological resources for effective self-exploration and commitment. In essence, support from others and psychological resources are two critical factors for an individual’s self-identity development. These factors are not mutually exclusive but are instead interrelated. According to cognitive psychology theory, the influence of any event on an individual must pass through a system of self-perception evaluation before influencing emotional perception and behavioral outcomes. Support received from significant others can be evaluated by the individual’s self-perception system, and subsequently internalized as a valuable psychological resource, thereby effectively alleviating negative emotions such as hopelessness. Previous studies have shown that social support perceived by college students, as an external factor, can alleviate depression and hopelessness triggered by the stress of life events (Wang et al., 2022).

**Figure 1:**
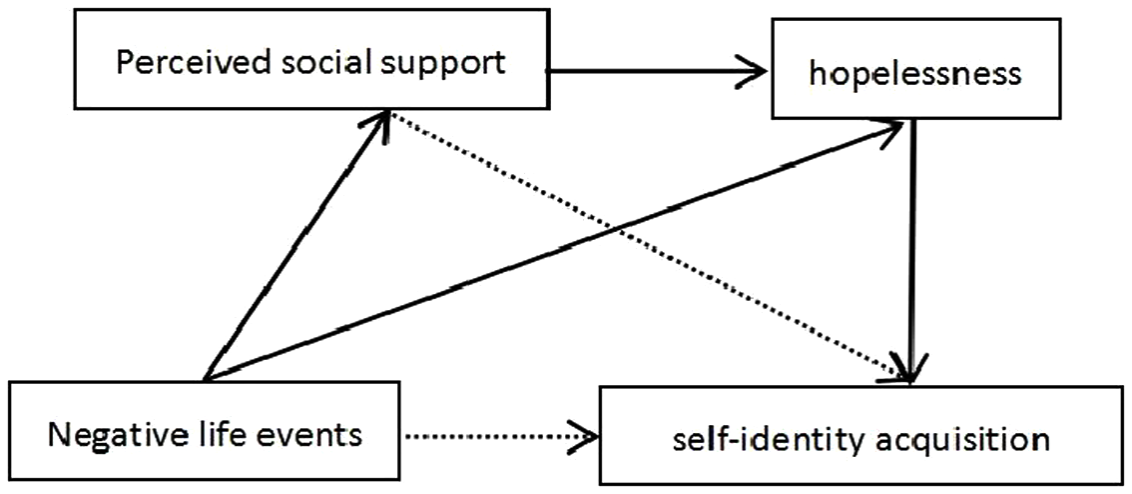
Hypothesized conceptual model of the chain mediation.

The attachment theory holds that the quality of parent–child relationships significantly influences an individual’s understanding of the social and emotional domains, serving as a crucial determinant in their emotional growth and the development of self-identity (Wang et al., 2007). Studies focusing on adolescents from families where mothers have abandoned them have revealed that these individuals may experience more severe psychological conflicts and pressures compared to their peers who receive maternal care and support over time. Such adverse feelings subtly hinder their self-identity development and may even result in distortion (Gao, 2022). Conversely, individuals from intact family structures tend to perceive greater familial support, which is more conducive to constructing their cognitive system, forming positive cognition and internal self-evaluation (Wang et al., 2022; Xie, 2017). And it facilitates the achievement of a cohesive self-identity (Becht et al., 2017). Therefore, we posit that external support can mitigate negative emotions such as hopelessness among college students. Finally, we propose hypothesis 4 (H4): perceived social support and hopelessness play a chain mediating role between negative life events and college students’ self-identity, as depicted in the mediation model hypothesis (refer to Figure 1).

## 2 Materials and methods

### 2.1 Sampling method and participants

This study used data from four universities in Jiangxi and Zhejiang provinces of China, to investigate the impact of negative life events on self-identity acquisition among college students who have experienced being left behind and/or migration. A simple convenient sampling was used in the data collection. Taking the class as a unit and with the informed consent of the students, relevant teachers used a unified filling guidance. The students completed the questionnaires independently and handed them in on the spot. A total of 1,240 valid questionnaires were returned out of 1,295 distributed, resulting in a valid response rate of 95.75%. The criteria for selecting college students with left-behind experiences are as follows: (1) individuals must have lived apart from one or both parents who were away for work, business, etc.; (2) before the age of 16; (3) this separation must have lasted more than 6 months. Migrant children refer to those under the age of 16 who have relocated from their registered residence and accompanied one or both of their parents to urban areas for over 6 months (National Women's Federation Research Group, 2013). It is noteworthy that some individuals had both left-behind and migrant experiences before the age of 16. Therefore, participants in this survey were divided into four groups: the left-behind-migrant group (Group 1), the left-behind group (Group 2), the migrant group (Group 3), and the no left-behind or migrant experiences group (i.e:, the normal group, Group 4). A total of 180 students were in Group 1, who had both left-behind and mobility experiences before the age of 16; there were 556 students in Group 2, who had stayed at home before 16 years old but did not migrate; the Group 3 consisted of 117 students with migration experience but no history of being left behind; there were 387 students in group 4, who had neither migrant nor left-behind experiences before reaching age 16. Among all respondents, there were 452 boys and 788 girls. The average age was (19.84 ± 0.96) years old. This study was conducted with a commitment to ensuring the utmost honesty and impartiality in all research procedures. The researchers adhered to ethical standards set by Yichun University. The survey contents were informed with the written consent of the participants.

### 2.2 Measures

#### 2.2.1 College students self-rating life events checklist

The List, developed by Liu et al. (1997) and revised by Zhang (2012), is primarily utilized to assess the level of distress caused by negative life events in the past 12 months. It consists of 32 items. For instance, the degree to which participants experienced psychological symptoms following instances of being treated with indifference or discriminated against. Responses are rated on a 6-point scale (0 = Not happening; 1 = No impact whatsoever; 2 = Mild impact; 3 = Moderate impact; 4 = Heavy impact; 5 = The impact is extremely severe). This scale yields a composite score, with higher scores indicating a greater adverse effect of negative life events. The internal consistency for this study was found to be satisfactory (*α* = 0.804).

#### 2.2.2 Self-identity acquisition (SIA)

This study utilized the Self-identity acquisition (SIA) subscale from the Chinese Simplified version of the Extend Objective Measure of Ego Identity Status-2 in College Students, as revised by Wang and Chen (2013). The SIA is an 8-item self-report tool to assess the degree of self-identity acquisition, for example, “It took me a long time to decide what I should do, and now I have a clear career direction.” Responses are rated on a 6-point scale ranging from 1 (very inconsistent) to 6 (very consistent), with scores totaling between 8 and 48. Higher scores indicate greater levels of self-identity acquisition. According to Jones et al. (1994), the classification point of self-identity status is M + SD/2. The score of the self-identity acquisition higher than M+ (SD/2) can be judged as the state of self-identity acquisition. The subscale demonstrated good internal consistency reliability at 0.73.

#### 2.2.3 Perceived social support scale (PSSS)

The PSSS revised by Jiang in 2001 (Chen et al., 2016), consists of 12 questions divided into three dimensions: support from family, support from friends, and support from other aspects. It primarily serves to assess individuals’ subjective perceptions and evaluations of external assistance. For instance, the extent to which participants felt that “my friends can provide meaningful support to me.” Responses are scored on a 7-point Likert scale ranging from 1 to 7. The PSSS produces a composite score, with higher scores indicating greater perceived support. In light of the specific context of college students, the terms “leaders, relatives, and colleagues” have been modified to “teachers, relatives, and classmates” for this study. The PSSS had good internal consistency (*α* = 0.90).

#### 2.2.4 Hopelessness

This study utilized the hopelessness subscale of the Self-rating Idea Of Suicide Scale (SIOSS) developed by Xia et al. (2002). The hopelessness subscale is primarily employed to assess the level of despair in life. It consists of a 12-item questionnaire, with participants reporting whether certain conditions occurred in the past 2 weeks, such as “I wanted to end my life.” Response options for each item are scored as “0 = never” or “1 = yes.” The total score, ranging from 0 to 12, is computed by summing responses across all 12 items; with higher scores indicating greater levels of despair. Additionally, this study demonstrated a high internal consistency for the subscale (α = 0.79).

### 2.3 Data analyses

T-tests and one-way ANOVA were employed to compare the scores and differences in self-identity acquisition among the four groups of college students. Next, Chi-square tests were conducted to assess whether there was a distribution equilibrium of the number of college students with different experiences who obtained self-identity acquisition. This was followed by Pearson correlational analyses examining the associations between the variables. Subsequently, a chain mediation analysis was performed following the method described by Hayes to explore whether perceived social support and hopelessness mediate the relationships between negative life events and self-identity acquisition. Bootstrap resampling with 5,000 samples and a percentile estimate of the confidence interval of the direct, indirect, and total effects were employed for this analysis.

### 2.4 Common method bias test

A common method bias test was conducted to examine the variables involved in the study. The findings indicated that there was no significant common method bias in this study when more than one factor was extracted, and the variance of the first factor explained 27.029% of the total variance, which is less than 40%.

## 3 Results

### 3.1 Acquisition of self-identity among four groups of college students

The scores for self-identity acquisition among the left-behind-migrant group (Group 1), the left-behind group (Group 2), the migrant group (Group 3), and the no left-behind or migrant group (Group 4) were 28.25 ± 9.13, 30.88 ± 6.22, 31.67 ± 6.97, 32.48 ± 6.04, respectively. Results of the one-way ANOVA indicated a significant main effect of the different groups on the acquisition of self-identity (*F* = 16.665, *p* < 0.001, *η*^2^ = 0.039). Pairwise comparisons revealed that Group 1 exhibited the lowest score in self-identity acquisition, which was significantly lower than those of the other three groups (*p* < 0.001). Conversely, Group 4 achieved the highest score, which was significantly higher than both Group 1 (*p* < 0.001, *η*^2^ = 0.071) and Group 2 (*p* < 0.001, *η*^2^ = 0.016). Additionally, the score of Group 2 was significantly lower than that of Group 4 but higher than that of Group 1 (*p* < 0.001, *η*^2^ = 0.025). No significant differences were observed between Group 3 and either Group 4 or Group 2 (*p* > 0.05).

It was discovered that a total of 329 participants (26.5%) were in a state of self-identity acquisition. Among these, the proportion of students who had achieved self-identity was the lowest in Group 1 (6.67%), followed by Group 2 (22.3%), Group 3 (36.75%), and finally, Group 4 (38.76%). Furthermore, a *χ*^2^ test [4 (different experiences)*2 (whether the status of self-identity acquisition or not)] revealed an uneven distribution among college students achieving self-identity (*χ*^2^ = 77.50, df = 3, *p* < 0.001). It was also noted that students with both left-behind and migrant experiences were less likely to be in a state of self-identity achievement compared to those in the other three groups.

### 3.2 Correlation analysis

The correlation coefficients among the study are presented in Table 1. Pearson’s correlations revealed that the four variables were significantly correlated with each other in the two groups that experienced being left behind. However, no significant relationships were found between self-identity acquisition and either negative life events or hopelessness in the no left-behind or migrant group and the migrant group. Additionally, in the no left-behind or migrant group, negative life events did not show a significant correlation with perceived social support.

**Table 1:** Correlation analysis for each variable.

Group	Variables	1 Negative life event	2 Perceived Social Support	3 Hopelessness	4 self-identity acquisition
No left-behind or migrant group	1	1	−0.05	0.37^***^	−0.05
	2		1	−0.11^*^	0.13^**^
	3			1	−0.04
	4				1
Both left-behind and migrant group	1	1	−0.22^**^	0.54^***^	−0.39^***^
	2		1	−0.44^***^	0.38^***^
	3			1	−0.65^***^
	4				1
Left-behind group	1	1	−0.10^*^	0.24^***^	−0.11^*^
	2		1	−0.36^***^	0.35^***^
	3			1	−0.23^***^
	4				1
Migrant group	1	1	−0.2^*^	0.24^**^	−0.16
	2		1	−0.19^*^	0.22^*^
	3			1	−0.17
	4				1

Significance level: *p < 0.05; **p < 0.01; ***p < 0.001.

### 3.3 Meditation effect

**Figure 2:**
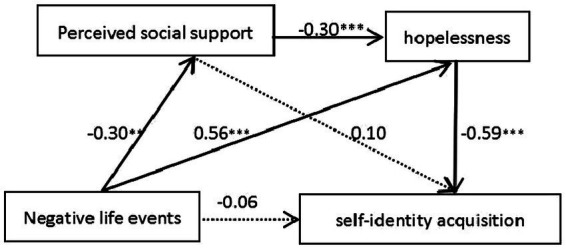
Both left-behind and migrant group. *p < 0.05; **p < 0.01; ***p < 0.001.

**Figure 3:**
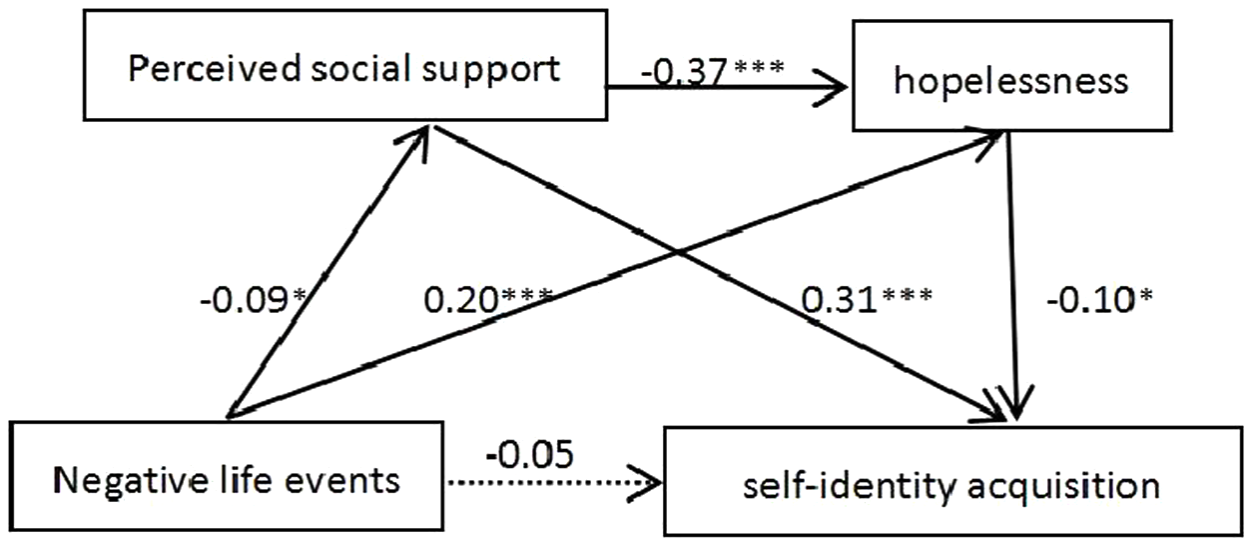
Left-behind group. *p < 0.05; **p < 0.01; ***p < 0.001.

According to the correlation analysis, no significant relationships were found between the dependent variable and either independent variable or mediating variable (M2) in the no left-behind or migrant group and the migrant group. Only when there is a high degree of correlation between the variables, it makes sense to conduct a regression analysis to seek the specific form of correlation. But the data from the left-behind-migrant group and the left-behind group met the conditions for conducting a mediation test. In this study, self-identity acquisition was taken as the dependent variable, negative life events as the independent variable, and perceived social support along with hopelessness as the mediator variables. The PROCESS program (Hayes, 2013) developed by Hayes was utilized to conduct the chain mediation effect for the students in these two groups. The regression tests revealed that the negative life events had a detrimental impact on self-identity acquisition for both the left-behind-migrant group (standard path estimate = −0.39; *p* < 0.05) and the left-behind group (standard path estimate = −0.11; *p* < 0.05). However, upon incorporating the two mediating variables—perceived social support and hopelessness, the direct effects of negative life events became statistically insignificant in both groups (*p* > 0.05). These findings are presented in Table 2 and Figures 2, 3.

**Table 2:** The effect relationship between the factors.

			Group 1					Group 2				
Variable			Fitting index			Beta coefficients		Fitting index			Beta coefficients	
			*R*	*R^2^*	*F*	*β*	*t*	*R*	*R^2^*	*F*	*β*	*t*
Self-identity acquisition	←	Negative life events	0.39	0.15	31.27	−0.39	−5.59 ^***^	0.11	0.01	6.55	−0.11	−2.56^*^
Perceived social support	←	Negative life events	0.22	0.05	9.32	−0.30	−3.05 ^**^	0.11	0.01	6.16	−0.09	−2.48 ^*^
Hopelessness	←	perceived social support	0.63	0.40	59.65	−0.30	−5.66 ^***^	0.41	0.17	56.47	−0.37	−8.67 ^***^
	←	Negative life events				0.56	7.85^***^				0.20	5.21^***^
Self-identity acquisition	←	Perceived social support	0.66	0.43	45.14	0.10	1.75	0.37	0.14	28.88	0.31	7.17^***^
	←	hopelessness				−0.59	−7.84^***^				−0.10	−2.47^*^
	←	Negative life events				−0.06	−0.74				−0.05	−1.24

Group 1:both left-behind and migrant group; group2:left-behind group. *p < 0.05; **p < 0.01; ***p < 0.001.

The Bootstrap method of deviation-corrected non-parametric percent position confidence interval was used to investigate the simple mediated effect and the chain mediated effect in this study. As depicted in Tables 3, 4, in the left-behind-migrant group, negative life events hurt self-identity acquisition through the simple mediating effect of hopelessness as well as the chain-mediated effect involving perceived social support and hopelessness. The finding indicated that the mediating effects along these two pathways were significant. However, it was found that the indirect effect of perceived social support did not reach statistical significance, as the Bootstrap 95% confidence interval included 0. Nevertheless, the total indirect effect proved to be significant, with a mediation proportion of 87.08%.

**Table 3:** Analysis of the mediating effect between negative life events, perceived social support, hopelessness, and Self-identity acquisition in group 1.

Intermediary path	Meditation effect	BootSE	95%CI		Proportion of mediated effect
			Lower limit	Upper limit	
Total indirect effect	−0.414	0.069	−0.550	−0.280	87.08%
Path 1	−0.030	0.020	−0.075	0.003	
Path 2	−0.331	0.058	−0.449	−0.223	69.52%
Path 3	−0.053	0.026	−0.112	−0.011	11.19%

Path 1: Negative life events → perceived social support → self-identity acquisition; Path 2: Negative life events → hopelessness → self-identity acquisition; Path 3: Negative life events → perceived social support → hopelessness → self-identity acquisition. The same as the following.

**Table 4:** Analysis of the mediating effect between negative life events, perceived social support, hopelessness, and Self-identity acquisition in group 2.

Intermediary path	Meditation effect	BootSE	95%CI		Proportion of mediated effect
			Lower limit	Upper limit	
Total indirect effect	−0.053	0.018	−0.091	−0.019	53.23%
Path 1	−0.029	0.013	−0.058	−0.005	29.54%
Path 2	−0.020	0.010	−0.042	−0.003	20.16%
Path 3	−0.004	0.002	−0.008	−0.002	3.53%

Within the left-behind group, it was observed that both the simple mediated effects of perceived social support and hopelessness were significant. Additionally, the chain- mediated effect was demonstrated to be significant as well. The total indirect effect within this group was considered significant, with a proportion of mediated effect at 53.23%.

## 4 Discussion

China places great importance on “root” culture. At the national level, “root culture” refers to the spiritual core of the Chinese nation and serves as a source of strength for its dynamic development. For an individual, it signifies a return to one’s “home.” Young adults leave their hometowns for work primarily to earn a living, often leaving their children behind in their native places, resulting in an increasing number of left-behind children. Secondly, there exists a tradition of family mutual assistance within Chinese culture. The spirit of familial support is deeply ingrained in Chinese values; it is considered natural for parents to assist their children. As noted by Liu (2015), “the duty of parents to help their children is also natural.” Parents willingly assume the responsibility of caring for their grandchildren, enabling them to work outside without distractions—thus contributing further to the phenomenon of left-behind children.

In recent years, with the implementation of national education policies aimed at supporting left-behind children and the improved economy for young adults, there has been a notable rise in migrant children. Some among these migrant youth have previously experienced being left behind; consequently, there are now instances where children possess both left-behind and migrant experiences.

The results of this study revealed that students with left-behind experiences exhibited poorer self-identity acquisition compared to their peers without such experiences. Furthermore, those who have experienced both left-behind and mobility demonstrated the lowest levels of self-identity acquisition. Additionally, this study delved into the relationship and internal mechanism of negative life events, self-identity acquisition, perceived social support, and hopelessness within the two groups of students who have been left behind. The results showed that perceived social support and hopelessness played a chain mediating role in the influence of negative life events on self-identity acquisition. This research contributes to a more comprehensive understanding of the factors affecting self-identity acquisition among students who have experienced being left behind and elucidates how these factors are interconnected within the process of self-identity acquisition.

### 4.1 Self-identity acquisition among students with left-behind and/or migrant experiences

The findings of the current study were consistent with the previous related research (Xie, 2017), indicating that the status of self-identity acquisition among college students in the left-behind-migrant group was the worst compared to the other three groups. The students who have experienced both being left behind and mobility encountered separation from their parents and familiar environments as they transitioned into new surroundings. This dual shift in both internal and external family environments makes these individuals more prone to confusion regarding their self-cognition during the process of self-identity development. They are prone to giving rise to questions such as” Who I am “and “What I can be,” etc. As a result, it is difficult for them to make positive and firm self-commitments based on hard exploration. Additionally, the level of self-identity acquisition among college students in the left-behind group was lower than that of their peers in both the no left-behind or migrant group and the migrant group (Xie, 2017). living with guardians instead of parents may contribute to incomplete family functioning, leading to limited parent–child interaction experiences and diminished psychological and emotional support from parents. These factors are detrimental to the development of individual self-identity.

### 4.2 Negative life events and self-identity acquisition

Negative life events were found to have a negative correlation with self-identity acquisition in both the left-behind-migrant group and the left-behind group. It was observed that negative life events could effectively predict self-identity acquisition for both groups of students, which confirmed the results of a previous study on general college students (Wang, 2019). This suggests that experiencing more negative life events is a significant factor influencing the acquisition of self-identity among college students. When these individuals encounter negative events in their academic or interpersonal relationships during college, they are more prone to encountering trauma and frustration. Consequently, this may lead them to doubt and deny themselves (Yang, 2017), resulting in issues related to identity identification and self-value. However, when the variables of perceived social support and hopelessness were included, the predictive power of negative life events on the acquisition of self-identity for both groups became insignificant. Simultaneously, there was a notable increase in the explanatory power (△R2) of the equation concerning self-identity acquisition. This suggests that while negative life events are important factors indirectly influencing self-identity acquisition through other variables, their direct predictive power diminishes when additional contextual elements are considered.

### 4.3 The mediating role of perceived social support

Perceived social support was found to have a negative correlation with negative life events and a positive correlation with self-identity acquisition in both the left-behind-migrant group and the left-behind group. These findings were consistent with previous research results (Zhou et al., 2022). The results of the mediation test indicated that perceived social support played a mediating role between negative life events and self-identity acquisition within the left-behind group; however, no significant mediating effect was observed in the left-behind-migrant group.

Negative life events have been shown to foster negative emotions, undermine social support systems (Gao, 2017), and reduce awareness of external support. According to the social support theory (Huber et al., 2021), such support serves as an important protective factor. Robust social support, parental care, and educational investment can mitigate the detrimental effects of adverse life experiences on individuals, facilitate the formation of self-identity acquisition, and assist individuals in achieving the unity of the past selves, present reality selves, and ideal selves. Previous studies have demonstrated that compared to the no left-behind or migrant group, individuals from the left-behind group, the migrant group, and the left-behind-migrant group faced challenges in social adaptation (Fan et al., 2009). They were prone to experience lower self-esteem along with mental health issues and difficulties in social adjustment (Xie et al., 2015). Nevertheless, mobility experiences may also serve as a beneficial counterbalance to these negative impacts (Zhang et al., 2015).

For college students in the left-behind-migrant group, experiencing mobility fosters a greater connection to urban civilization. Notably for this group, there is no shortage of parental companionship; and parents provide additional emotional support and attention regarding education and daily life. These positive effects could directly enhance their access to high-quality educational resources as well as favorable educational assistance within urban settings. Thus, this support buffers the adverse impact of being left behind, promotes positive behaviors, bolsters resilience in the face of negative life events, and improves the development of self-identity. Specifically, this group possesses an inherently higher perception of societal and family support. As a result, the buffering effect of perceived social support between negative life events and the acquisition of self-identity is relatively diminished. In contrast, individuals who have solely experienced being left behind tend to have a lower perception of support from family and others (Xie et al., 2015). Upon entering university, these left-behind individuals who face challenges stemming from negative life events, find that support from friends and family becomes increasingly essential. This support can serve as a mediating factor in alleviating the detrimental effects associated with negative life events while enhancing their likelihood of utilizing social resources for problem-solving (Xie, 2017), ultimately promoting the formation of self-identity (Quan et al., 2008).

### 4.4 The mediating role of hopelessness

The results showed that hopelessness exhibited a negative correlation with self-identity acquisition and a positive correlation with negative life events. Furthermore, hopelessness was found to play an intermediating role in the relationship between negative life events and self-identity acquisition within both the left-behind-migrant group and the left-behind group. These results aligned with previous investigations into ordinary college students (Xie et al., 2017).

Negative events such as parental absence, poor parent–child relationships during early experiences of being left behind, and academic pressure during college, all are prone to contribute to adverse self-cognition among students (Yi and Zhang, 2018). When individuals are unable to effectively cope with these negative life events or internalize them as stable emotional factors, they are more susceptible to feelings of anxiety, depression, and despair. Such emotions often manifest as pessimistic attitudes toward their abilities and prospects. It is important to note that the formation of self-identity acquisition is not given but rather attained through effort throughout personal growth processes (Hasanshahi et al., 2018). Once feelings of hopelessness are triggered by negative life events, individuals tend to lower their expectations for themselves and the future. This tendency can hinder their ability to maintain a positive and proactive stance toward personal growth and development in the process of “becoming” such as engagement in various affairs.

Moreover, the mediation effect of hopelessness between negative life events and self-identity acquisition was found to be more pronounced in the left-behind-migrant group compared to the left-behind group. This suggests that there exists a greater indirect impact of hopelessness between negative life events and self-identity acquisition within the left-behind-migrant group. In comparison with students in the left-behind group, those in the left-behind-migrant group have more transient experiences. On the one hand, their experiences of being left behind have a significant negative impact on their self-adjustment capabilities and self-psychological resilience when confronted with negative life events, leading to increased feelings of loneliness, depression, and anxiety (Zhang et al., 2019). On the other hand, according to social comparison theory (Festinger, 2016), when these individuals relocate to unfamiliar cities, they often exacerbate their dissatisfaction by comparing their environment, identity, and abilities with those of others. As a result, they need to constantly adjust their self-cognition and behaviors during the process of “becoming” as they strive to integrate into city life. However, due to cultural differences, lifestyle distinctions, and social discrimination, it becomes increasingly challenging for them to assimilate into urban life and establish social relationship networks. This makes it easier for them to feel lonely, anxious, and hopeless. Consequently, there is a decline in core self-evaluation and self-esteem from the present self to the future self, ultimately leading to the development of a negative self-identity state.

### 4.5 The chain mediating role of perceived social support and hopelessness

Perceived social support and hopelessness played a chain mediating role in the influence of negative life events on self-identity acquisition among both the left-behind-migrant group and the left-behind group. This finding verified the stress-buffering model of social support (Huber et al., 2021).

While perceived support is a psychological reality, it can be considered as an actual variable that influences individual behavior and development. Moreover, perceived social support has been shown to predict individuals’ physical and mental well-being more effectively than actual received support (Quan et al., 2008), thereby impacting the development of self-identity. Individuals with a higher perception of social support tend to evaluate negative life events more positively, which is beneficial for lowering the level of hopelessness. Furthermore, those who perceive greater levels of support demonstrated more positive exploration and commitment in their development of self-identity, ultimately facilitating the formation of self-identity acquisition.

Hopelessness was found to play an intermediary role in the influence of perceived social support on self-identity acquisition within the two groups. It was observed that increased perceptions of social support could enhance the acquisition of self-identity by reducing feelings of hopelessness in the left-behind-migrant group. This showed that for the students in this group, hopelessness exerts a more direct impact on their self-identity acquisition compared to perceived social support. In contrast, for students belonging to the left-behind group, negative life events were found to impact self-identity acquisition through the separate and chain mediating effects involving perceived social support and hopelessness. These findings revealed the internal mechanism of negative life events on the acquisition of self-identity among the two groups of left-behind students.

Specifically, it was observed that students from both groups tended to reduce their use of support when confronted with negative emotions such as hopelessness following adverse life experiences. This behavior proved detrimental to individuals’ active exploration of identity, future goals, and other aspects; furthermore, it did not promote positive and firm self-commitment. According to the Resource Conservation Theory (Lin et al., 2018), the extent to which negative life events hinder college students from establishing their self-identity is, to a significant degree, contingent upon whether the resources they acquire can be maintained in a balanced state. Among these resources, family and social support play a pivotal role in enhancing individual resilience against stressors. When an individual is under the pressure of an overwhelming negative life event, bolstering their sense of support can mitigate its adverse effects through the chain mediation process. This can facilitate the adoption of positive coping strategies, reduce feelings of hopelessness, strengthen confidence in one’s abilities and prospects, and ultimately promote the development of self-identity.

### 4.6 Research significance

This research explored the self-identity acquisition among college students who have experienced being left behind or/and mobility. While previous studies have indicated that the influence of childhood experiences of being left behind or migrant on individuals persists into adulthood, there remains a relative scarcity of research focused on self-identity in college students with both left-behind or/and migrant experiences. This study contributes to the advancement of relevant policies aimed at supporting the development of children with diverse experiences, thereby safeguarding their future growth and well-being.

The research findings indicated that perceived social support and hopelessness play significant roles in the self-identity development of the two types of college students with left-behind experiences. This implies the necessity for psychologists and educators to recognize the potential impact of social support from parents, teachers, peers, and others on these students’ self-identity formation. Interventions should be developed to mitigate feelings of hopelessness while simultaneously fostering their commitment to identity development. Firstly, from the perspectives of psychological counseling practice and the collaboration between families and educational institutions, it is essential for enhancing the self-identity of college students. During the psychological counseling process, guiding parents to increase their psychological and material support for students can be highly beneficial. Simultaneously, within the school environment, encouraging teachers to promote the establishment of positive peer relationships among college students can further strengthen their self-identity. Secondly, guiding students in managing their own emotions is essential. Despair and other negative emotions can significantly impact the process of self-identity acquisition. When students face negative events and pressures in their academic and lives, it is important to encourage them to seek support from family members, teachers, friends, and others. Additionally, promoting active participation in class activities and school affairs can help alleviate feelings of hopelessness while enhancing their confidence in their abilities and expectations for the future. This proactive approach ultimately contributes to a more effective acquisition of self-identity.

### 4.7 Research limitations and prospects

While this study provides both theoretical and empirical support for investigating the relationship between negative life events and self-identity acquisition, several limitations warrant attention in future research.

Firstly, this study has not taken into account the impact of specific negative life events on the development of self-identity. Negative life events, such as those related to academics, livelihood, health, and family, can exert diverse influences on college students’ attainment of self-identity. Therefore, in future research, investigating various kinds of negative life events could provide greater depth to our insights.

Secondly, this research employed a quantitative research approach for the investigation. In the future, it is advisable to consider incorporating qualitative research methods, such as interviews, to gain deeper insights into the experiences that may influence the development of college students’ self-identity throughout their growth process.

Thirdly, the cross-sectional design of the study limited the ability to conduct causal analysis regarding the relationship between variables. Future longitudinal studies could explore the dynamic nature of self-identity in adolescents with left-behind experiences across various developmental stages, including middle school, high school, and university.

Finally, the sample of this study is limited, comprising only 1,240 students from four schools across two cities in China. Consequently, it remains uncertain whether the findings can be generalized to students in other cities or various types of schools. Future research should consider expanding the sample size and conducting investigations in diverse regions or different categories of schools, to further examine the generalizability of the results.

## 5 Conclusion

This study explored the status of self-identity acquisition among four categories of college students, and the relationship between negative life events, perceived social support (PSSS), hopelessness, and self-identity acquisition among two groups with experiences of being left behind. Findings revealed the status of self-identity acquisition in the left-behind-migrant group was the worst, the left-behind group followed. Furthermore, negative life events were found to indirectly predict self-identity acquisition through the chain-mediating effects of PSSS and hopelessness within both the left-behind group and the left-behind-migrant group. In addition, for individuals who experienced both left-behind and migrant situations, there was no significant mediating effect of PSSS between negative life events and self-identity acquisition; rather, hopelessness served as a stronger mediator compared to that within the left-behind group. The results of the present study underline the significance of mitigating the occurrence of negative life events and feelings of hopelessness.

## Data Availability

The raw data supporting the conclusions of this article will be made available by the authors, without undue reservation.
